# Frozen-Core Analytical Gradients within the Adiabatic Connection Random-Phase Approximation from an Extended Lagrangian

**DOI:** 10.1021/acs.jctc.4c01731

**Published:** 2025-03-06

**Authors:** Jefferson E. Bates, Henk Eshuis

**Affiliations:** † Department of Chemistry and Fermentation Sciences, Appalachian State University, Boone, North Carolina 28608-2021, United States; ‡ Department of Chemistry and Biochemistry, Montclair State University, Montclair, New Jersey 07043, United States

## Abstract

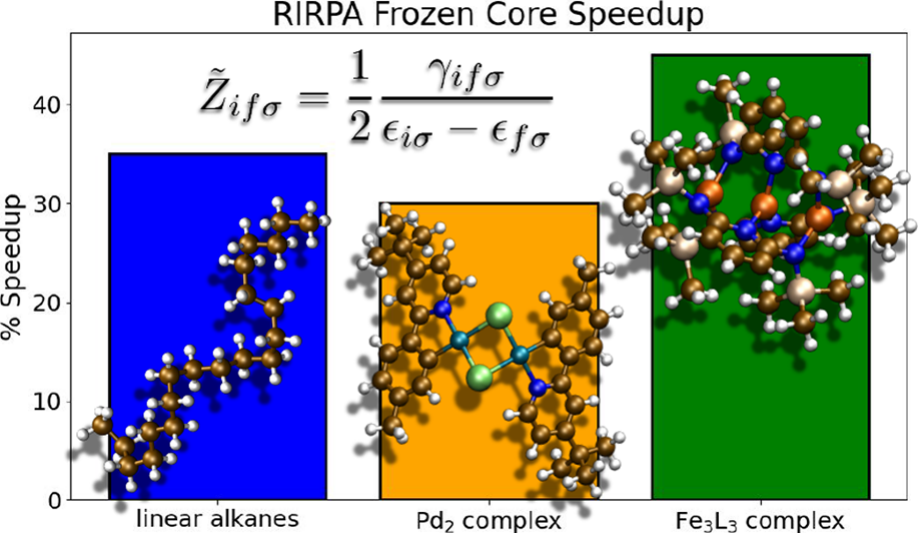

The implementation of the frozen-core option in combination with the analytic gradient of the random-phase approximation (RPA) is reported based on a density functional theory reference determinant using resolution-of-the-identity techniques and an extended Lagrangian. The frozen-core option reduces the dimensionality of the matrices required for the RPA analytic gradient, thereby yielding a reduction in computational cost. A frozen core also reduces the size of the numerical frequency grid required for accurate treatment of the correlation contributions using Curtis–Clenshaw quadratures, leading to an additional speedup. Optimized geometries for closed-shell, main-group, and transition metal compounds, as well as open-shell transition metal complexes, show that the frozen-core method on average elongates bonds by at most a few picometers and changes bond angles by a few degrees. Vibrational frequencies and dipole moments also show modest shifts from the all-electron results, reinforcing the broad usefulness of the frozen-core method. Timings for linear alkanes, a novel extended metal atom chain and a palladacyclic complex show a speedup of 35–55% using a reduced grid size and the frozen-core option. Overall, our results demonstrate the utility of combining the frozen-core option with RPA to obtain accurate molecular properties, thereby further extending the range of application of the RPA method.

## Introduction

The random phase approximation (RPA) method has become a popular electronic structure method within the adiabatic connection framework of density functional theory (DFT) for chemistry, physics, and materials science.^1−5^ RPA overcomes a number of shortcomings of semilocal functionals such as the elimination of self-interaction error in the exchange energy and natural treatment of long-range interactions. As a nonperturbative method, RPA can be equally applied to strongly correlated systems with a small HOMO–LUMO gap as it can to closed shell, main group systems. RPA has been shown to accurately predict reaction energies for a wide range of chemical reactions,^6,7^ binding energies for dispersion bound systems,^8^ and to be useful as a method to train advanced machine-learning based functionals.^9^ Results for transition metal systems,^10−16^ as well as for lanthanide systems,^17−19^ further indicate RPA’s applicability across the periodic table. Efficient implementations of RPA achieve comparable computational costs to hybrid functionals by employing a combination of resolution-of-the-identity (RI) and numerical time or frequency integrations.^20−27^ It has been shown that using a Kohn–Sham (KS) reference is key for obtaining accurate structural results.^28^ Implementations of analytic first- and second-order molecular properties have also been reported for RPA, which enable calculation of, e.g., optimized molecular structures, vibrational frequencies, and magnetic properties.^28−32^

As a consequence of using the fluctuation–dissipation theorem, pieces of the RPA energy and its analytic gradient depend on products of occupied and virtual orbitals. The number of these orbital products rapidly increases with basis set size resulting in high computational costs and limiting the applicability of RPA. A finite number of the occupied orbitals correspond to electrons in core orbitals which are known to have minimal impact on valence properties and can therefore be excluded from the correlated parts of the calculation.^33,34^ Removal, or “freezing”, of these orbitals in the correlation pieces leads to a potential reduction in computational effort proportional to the number of frozen orbitals. However, removal of the core orbitals from the correlation treatment requires nontrivial changes to the working equations for RPA implementations to distinguish between the frozen and active occupied orbitals. The frozen-core option is not limited to RPA and has been implemented for other correlated theories such as MP2^34−36^ and coupled cluster methods.^33,37−39^

In this work the combination of the frozen-core option with an extended Lagrangian to calculate first-order molecular properties for RPA is reported. The approach is based on our previously reported implementation of RIRPA gradients in the turbomole program package.^28^ Drontschenko et al. first used the frozen-core option in their implementation of RPA gradients which combines Cholesky decomposition techniques with elimination of perturbed MO coefficients from the analytic gradient.^30^ Very recently, Tahir et al. also reported an efficient method for RPA first-order properties using the frozen-core option.^32^ The main difference between these approaches and ours is that total RPA energy differences are evaluated from partial derivatives of an extended Lagrangian, thus completely avoiding the implicit dependence of the energy on various KS quantities. In addition, our approach is immediately applicable to open- and closed-shell systems.

When using Curtis-Clenshaw quadratures to evaluate the RIRPA correlation energy, a sensitivity measure is calculated that reflects the convergence of the numerical frequency integration.^20^ Keeping this value below 10^–4^ typically requires ∼30 frequency grid points for large gap systems, but could require up to 100 or more points for small-gap systems with the core electrons included. Utilizing the frozen-core option will be shown to reduce the number of grid points required to achieve a sufficiently small sensitivity measure, which is an additional factor contributing to the computational savings of this approach.

The paper is organized as follows. First, the theory for the RIRPA energy functional and extended Lagrangian are briefly reviewed, followed by a discussion of the partial derivatives and determination of the Lagrange multipliers. Next, optimized molecular geometries are reported for a set of main-group complexes, as well as geometries and vibrational frequencies for closed and open-shell first-row transition metal complexes. This is followed by a discussion of the computational speedup in the form of timing tests for the all electron and frozen-core algorithms. The paper ends with a conclusion.

## Theory

In the following we outline in detail the differences and similarities of the frozen-core implementation with the full-core implementation. Freezing a subset of occupied (core) orbitals impacts the algorithm for the implementation of the analytical derivatives for RPA because of the restricted sums over occupied orbitals in the correlation contributions. The use of a smaller occupied space limits the number of orbital products that need to be taken into account and yields a computational speedup. However, not all loops over occupied orbitals can be restricted and several parts of the algorithm are unaltered from the original full-core implementation.

The RPA gradient is done analogously to the RIMP2 frozen-core implementation by Weigend and Häser^35^ and we adopt the same convention for labeling orbitals, namely *a*, *b*, ··· refer to virtual orbitals, *f*, *g* to frozen occupied orbitals, *i*, *j*, *k* to active occupied orbitals, *l*, *m*, *n* to general occupied orbitals, and *p*, *q*, ··· to general molecular orbitals from all subspaces. Greek indices refer to quantities in the atomic orbital (AO) basis, however σ = α, β are used to denote the different spin components. Capital Roman letters *P*, *Q*, ··· refer to quantities in the auxiliary basis set, {η_*P*_}. The RPA gradient in this work is obtained through a “post-KS” approach that relies on a reference determinant generated from a semilocal functional. The dimensions of the various orbital subspaces will be denoted as *N*_s_ is the number of spins, *N*_occ_ is the total number of occupied orbitals, *N*_froz_ is the number of frozen occupied orbitals, *N*_act_ is the number of active occupied orbitals in the correlation treatment, *N*_virt_ is the number of virtual orbitals, and *N*_aux_ is the number of auxiliary basis functions. The needed equations are thoroughly laid out in ref (28). The same notation will be followed herein, though only key points are reviewed to contrast with the full-core implementation.

### Summary of the RIRPA Energy Functional

A spin-unrestricted reference determinant represented through its KS MO coefficient matrix, **C** = (**C**_α_, **C**_β_), satisfies the KS equations
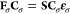
1where
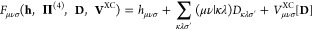
2is the one-particle effective KS Hamiltonian that depends on the one-electron Hamiltonian **h**, the four-index electron repulsion integrals (ERI) **Π**^(4)^, the spin-unrestricted one-particle density matrix **D**, and the adiabatic XC potential matrix **V**^XC^ calculated within the Handy-Neumann approximation.^40^ Mulliken notation is utilized for the ERIs throughout
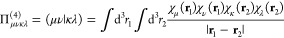
3where {χ_μ_} refer to atom-centered Gaussian basis functions. The MO coefficients are required to be **S**-orthonormal
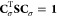
4where *S*_μν_ is the overlap of the nonorthogonal AO basis, ensuring the orthonormality of the MOs. With the reference determinant in hand, the total RIRPA energy can be computed as
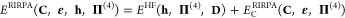
5where *E*^HF^ is the Hartree–Fock energy evaluated at the KS reference determinant (eq 9 in ref (28)) and *E*_C_^RIRPA^ is the RIRPA correlation energy.

An efficient implementation of these equations utilizes resolution-of-the-identity (RI) techniques^41,42^ to calculate an approximate factorization of the ERI supermatrix
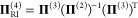
6where Π_μν *P*_^(3)^ = (μν|*P*) and Π_*PQ*_^(2)^ = (*P*|*Q*) are the 3-center and 2-center ERIs, respectively. The inverse of **Π**^(2)^ is calculated through a Cholesky decomposition, **Π**^(2)^ = (**Λ**^T^**Λ**), with **Λ** an upper-triangular matrix. This “Coulomb metric” approach^43,44^ leads to a variational upper bound for the RPA correlation energy and, in combination with imaginary frequency integration,^20^ yields an overall correlated method that scales as *N*^4^ log *N*, where *N* is a measure of system size.

Since the RIRPA energy depends on the MO coefficients and Lagrange multipliers **ε**, as well as parametrically on **h**, **Π**, and the AO and auxiliary basis functions, these parameters are gathered into a supervector, **X** = (**h**, **Π**^(4)^, **Π**^(3)^, **Π**^(2)^) leading to
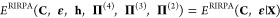
7**C** and **ε** are also parametrically dependent on the **V**^XC^ and **S** through eqs 1 and 4. Within these approximations the RIRPA correlation energy can be expressed entirely in the auxiliary basis as
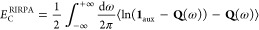
8where
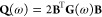
9and ⟨*A*⟩ indicates the trace of matrix *A*. The frequency-dependent supermatrix **G**(ω) and modified three-center integrals **B** are defined as
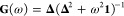
10
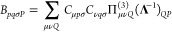
11**Δ** contains the virtual–virtual and occupied-occupied blocks of the Lagrange multiplier matrix, **ε**, which are not required to be diagonal. However, in the canonical KS orbital basis, Δ_*ia*σ*jb*σ*′*_ = (ε_*a*σ_ – ε_*i*σ_)δ_*ij*_δ_*ab*_δ_σσ*′*_ is diagonal and therefore so is **G**(ω). Furthermore, in a frozen-core calculation **Δ** and **B** only include the active occupied orbitals, reducing the dimensions to *N*_*s*_ × *N*_act_ × *N*_virt_ and *N*_*s*_ × *N*_act_ × *N*_virt_ × *N*_aux_, respectively.

Total differentiation of eq 7 with respect to a “perturbation parameter”, ξ, is straightforward, but does not lead to an efficient implementation due to the dense derivatives d**C**/dξ and d**ε**/dξ that appear. These derivatives would necessitate solving *f* coupled-perturbed KS (CPKS) equations for all *f* nuclear degrees of freedom if ξ represents, for example, a Cartesian nuclear displacement.^28^ To avoid this increase in computational expense, we follow Helgaker and Jo̷rgensen^45^ such that derivatives of MO coefficients and Lagrange multipliers are avoided entirely by defining an extended RIRPA energy Lagrangian.

### Extended RIRPA Energy Lagrangian

The RIRPA energy Lagrangian utilized is defined as^28^
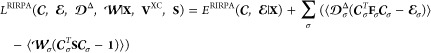
12which contains the independent variables , , , and , and is required to be stationary with respect to each of these. Though the RIRPA energy is not stationary at the KS reference orbitals due to its “post-KS” evaluation, the extended Lagrangian is stationary at this reference point. Thus,  and  are the Lagrange multipliers that ensure  and  satisfy the Kohn–Sham equations and the orthonormality constraints
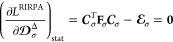
13
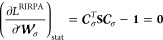
14Additionally, the Lagrangian is required to be stationary with respect to variations of  and  leading to two further equations that determine the remaining blocks of  and 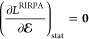
15
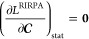
16At the stationary point, the Lagrange multipliers have all been fully determined leading to “stat =  = **C**,  = **ε**,  = **D**^Δ^, and  = **W**”, and the equivalence of the RIRPA energy and extended Lagrangian
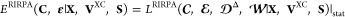
17Since the partial derivatives of *L*^RIRPA^ with respect to each Lagrange multiplier all vanish at the stationary point, first-order RIRPA properties can be immediately obtained as
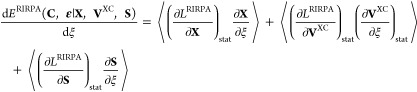
18thereby completely avoiding MO coefficient derivatives or derivatives of Lagrange multipliers.

### Evaluating the Lagrange Multipliers with Frozen-Core Electrons

Within RIRPA, correlation and orbital relaxation effects in the one-particle density matrix are captured by **D**^Δ^ which takes the following form in a frozen-core calculation
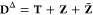
19In the MO basis, **T** accounts for the “unrelaxed” active occupied-occupied and virtual–virtual blocks. **Z̃** contributes the frozen occupied-active occupied block and **Z** contributes the full occupied-virtual block of **D**^Δ^, corresponding to orbital relaxation terms. To calculate **T**, the stationarity condition of the RIRPA Lagrangian with respect to the Lagrange multiplier , eq 15, leads to an equation for the active occupied-occupied block as well as the virtual–virtual block
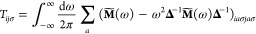
20
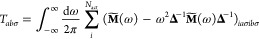
21The matrix **T** is constructed more readily compared to the full-core calculation since both **M̃** and **Δ** are restricted to active occupied orbitals. Additionally the frozen–frozen block of **T** is zero as a result. The symmetric supermatrix **M̃**, which is never explicitly constructed, is a function of matrix **Q** through **Q̃** as defined in eqs 31 and 32 of ref (28).
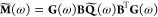
22
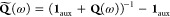
23

Due to the presence of the frozen core orbitals, **Z̃** is needed to account for frozen-active occupied orbital relaxation.^46^ Application of the **Z**-vector method^47^ results in a simple expression for **Z̃**, whose derivation is presented in Appendix A. The final result is that **Z̃** can be obtained analytically as
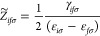
24without the need to solve a CPKS equation, where the nonsymmetric matrix **γ** is constructed as
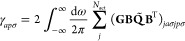
25
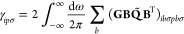
26Various blocks of **γ** contribute to the energy weighted density matrix, **W**, and also to the solution of the occupied-virtual **Z**-vector. Eq 25 has the same form as the full-core calculation, however the sum is only over active occupied orbitals within the frequency integration. In eq 26 the first index is restricted to the active occupied orbitals while the second remains unrestricted, leading to a slightly smaller object compared to the full-core case.

While **T** has reduced dimensions in the occupied-occupied block due to the frozen-core, the **Z**-vector spanning the occupied-virtual space requires all occupied orbitals (frozen and active) be included in its solution due to the one- and two-electron, frozen occupied orbital contributions that appear in the occupied-virtual block of **ε**_σ_^HF^ and its contribution to the gradient. Still, only a single solution of the CPKS equation in the occupied-virtual space with a modified right-hand side is required to obtain it,
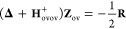
27The modified right-hand side for a frozen-core calculation can be expressed as
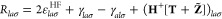
28where
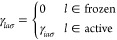
29Compared to the full-core calculation, the contribution from γ_*ia*σ_ has reduced dimension due to the frozen-core and the operator **H**^+^, eq 34 in ref (28), is now contracted with both **T** and **Z̃**.  has the same definition as in the full-core calculation, eqs 37 and 38 in ref (28), and therefore contains the same core contributions in both the full- and frozen-core calculations.

After solving for **Z**, the difference density due to correlation is constructed and used to calculate the Lagrange multiplier **W**. The various blocks of this matrix are obtained as
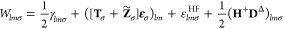
30
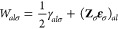
31
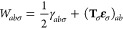
32While *W*_*lm*σ_ does not have reduced dimension in the frozen-core case, the ingredients used to build it all do, which results in a computational speedup compared to the full-core case. The occupied-occupied block in this case is constructed by patching together the frozen–frozen, frozen-active, and active–active contributions as discussed in Appendix A.

Utilizing these definitions and the stationarity of the RI-RPA Lagrangian, the final analytic gradient can be written in the AO basis using the same form as derived for the full-core calculation^28^
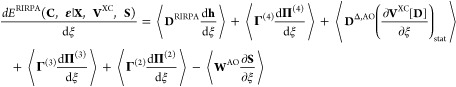
33The RIRPA one-particle density matrix, **D**^RIRPA^, and energy-weighted total spin one-particle density matrix, **W**^AO^, do not depend on the number or type of perturbations once the Lagrange multiplier **D**^Δ^ has been computed. In a frozen-core calculation
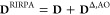
34
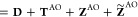
35with the AO transformed quantities
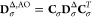
36
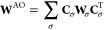
37The definitions for the four- (**Γ**^(4)^) and two-index (**Γ**^(2)^) relaxed two-particle density matrices, eqs 50 and 52 in ref (28), remain unchanged since they are expressed entirely in the AO and auxiliary basis, respectively. The definition for **Γ**^(3)^, eq 51 in ref (28), remains formally unchanged, however the sum over occupied orbitals is restricted to active occupied orbitals leading to a modest speedup compared to the full-core calculation. Compared to other implementations of frozen-core RPA gradients,^30^ our approach has the advantage that *total* RIRPA energy derivatives can be evaluated from the *partial* derivatives of an extended Lagrangian at its stationary point. This avoids all of the complicated implicit dependence of the RIRPA energy on **C**, **ε**, **X**, **V**^XC^, and **S**. Our method is also immediately applicable to both closed- and open-shell systems as demonstrated below.

### Implementation

Figures S1 and S2 in the Supporting Information outline the details of the frozen-core implementation. The same approximations and methods used for the full-core case can be immediately applied to the frozen-core calculation, such as the integral-direct, iterative subspace solution of the **Z**-vector equation,^48,49^ the RI-J approximation,^44,50^ and Schwarz screening of the ERIs.^51^ Step 6 in Figure S1 still remains the overall rate-limiting step, with an asymptotic scaling of .^28^ The current algorithm avoids four-index quantities through frequency integration making it comparable to low-scaling implementations of RIMP2 gradients.^35,52^ Instead, three-index quantities are needed implying that the construction and storage of *B*_*ia*σ *P*_ and Int2GBQt_*ia*σ *P*_, step (4) and (6) in Figure S1 respectively, are still the most intensive. Since these quantities are now expressed in the active occupied space, the required storage scales as  instead of  in the full-core case. An overview of the dimensionality of a subset of most relevant objects is provided in Table 1. The first-order properties calculated in this work have been implemented in a developer’s version of the turbomole program package^53^ and will be released in version 7.10.

**Table 1:** Overview of Symbols Used and Their Respective Dimensions in the Frozen- and Full-Core Calculations

(super) matrix	eq	frozen-core	full-core
**T**_oo_	20	*N*_s_ × *N*_act_ × *N*_act_	*N*_s_ × *N*_occ_ × *N*_occ_
**Z̃**	24	*N*_s_ × *N*_act_ × *N*_froz_	
**Z** and **R**	27 and 28	*N*_s_ × *N*_occ_ × *N*_virt_	*N*_s_ × *N*_occ_ × *N*_virt_
**B**_ov_	11	*N*_s_ × *N*_act_ × *N*_virt_ × *N*_aux_	*N*_s_ × *N*_occ_ × *N*_virt_ × *N*_aux_
**B**_oo_	11	*N*_s_ × *N*_act_ × *N*_occ_ × *N*_aux_	*N*_s_ × *N*_occ_ × *N*_occ_ × *N*_aux_
**G** and **Δ**	10		
**γ**_oo_	26	*N*_s_ × *N*_act_ × *N*_occ_	*N*_s_ × *N*_occ_ × *N*_occ_
**γ**_ov_	29	*N*_s_ × *N*_act_ × *N*_virt_	*N*_s_ × *N*_occ_ × *N*_virt_

## Results and Discussion

### Computational Details

The RPA calculations reported herein were based on self-consistent KS orbitals obtained with the PBE semilocal functional,^54^ unless otherwise stated. For the self-consistent field calculations, large integration grids (size 5) were used to converge the change in ground state energy and one-particle density matrices to at least 10^–7^. The number of frequency grid points used for RIRPA varies from system to system, but was always chosen to ensure a sensitivity measure of at least 1 × 10^–4^. The Karlsruhe def2-SVP, def2-TZVP, and def2-QZVP basis sets^55^ were used for many of the results presented below, however other basis sets were also utilized to ensure a direct comparison to the literature could be made. The corresponding auxiliary basis sets were used throughout^56,57^ in combination with the RI-J approximation for the two-electron Coulomb integrals.^58^ The default set of frozen-core orbitals in turbomole was used unless otherwise noted.

### Main-Group Complexes

To directly compare our implementation to the one by Drontschenko et al.^30^ we computed bond lengths and bond angles for the same set of small, main-group molecules using the same basis set, aug-cc-pwCVQZ.^59^ The results are shown in Table 2. The mean absolute deviation in bond lengths and angles between full-core and frozen-core RPA is 0.17 pm and 0.30 degrees, respectively, a difference that is small and comparable to what was reported in refs (30). and (32). For this small set, RPA bond lengths slightly increase on average when freezing core electrons, whereas bond angles slightly decrease.

**Table 2:** Comparison of Full-Core and Frozen-Core RPA Bond Lengths and Bond Angles for a Set of Small Main-Group Molecules, Taken from Ref (30)^a^

molecule	**full** distance	angle	**fc** distance	angle	**diff.** distance	angle
CH_4_	108.99		109.12		0.13	
Cl_2_	202.54		201.85		–0.69	
CO	113.54		113.77		0.23	
CS	154.44		154.72		0.28	
F_2_	144.39		143.88		–0.51	
C_2_H_2_	(C–H) 106.45		106.56		0.11	
	(C–C) 120.71		120.95		0.24	
CH_2_O	(C–H) 110.30	(H–C–O) 121.69	110.43	121.70	0.13	0.01
	(C–O) 121.15	(H–C–H) 116.61	121.32	116.60	0.17	–0.01
H_2_O	96.33	103.94	96.40	103.84	0.07	–0.10
H_2_O_2_	(O–H) 96.81	(O–O–H) 99.36	96.89	99.34	0.08	–0.02
	(O–O) 147.45	(H–O–O–H) 113.90	147.54	113.20	0.09	–0.70
H_2_S	133.86	92.18	134.05	92.18	0.19	0
HCl	127.76		127.86		0.10	
HCN	(C–H) 106.79		106.92		0.13	
	(C–N) 115.87		116.08		0.21	
HF	92.29		92.33		0.04	
HOCl	(O–H) 96.93	102.20	96.98	102.22	0.05	0.02
	(O–Cl) 171.91		171.57		–0.34	
N_2_	110.36		110.55		0.19	
NH_3_	101.62	106.13	101.73	105.97	0.11	–0.16
PH_3_	141.46	93.36	141.77	93.38	0.31	0.02
SiH_2_	151.21	93.72	151.82	92.47	0.61	–1.25
SiH_4_	147.55		147.99		0.44	
SiO	151.92		152.27		0.35	
				ME	0.11	–0.22
				MAE	0.17	0.30
				max	0.69	1.25

a Distances are reported in pm, angles in degrees. The basis set used is aug-cc-pwCVQZ. Included are the mean error (ME), the mean absolute error (MAE), and the absolute maximum error (max). The difference (diff.) is calculated as frozen-core (fc) minus full-core (full).

In addition to yielding similar geometries, the vibrational frequencies and dipole moments calculated using the frozen-core option are also very similar to their full-core counterparts for a selected set of small molecules. Numerical results can be found in the Supporting Information (see Tables S1 and S2). Dipole moments are minimally impacted by the frozen-core option, typically differing by less than 0.01 debye. Vibrational frequencies show larger deviations between full- and frozen-core calculations, with the impact on small molecules being the most pronounced, however the relative shifts are still less than 1–2% of the vibrational frequency. For instance, the *a*_1_ and *b*_1_ stretching modes predicted for H_2_O are approximately 40 cm^–1^ smaller with a 1*s* frozen core, but this is only a 1% error compared to the all electron results.

### Transition Metal Complexes

#### First-Row Transition Metal Complexes

**Figure 1:**
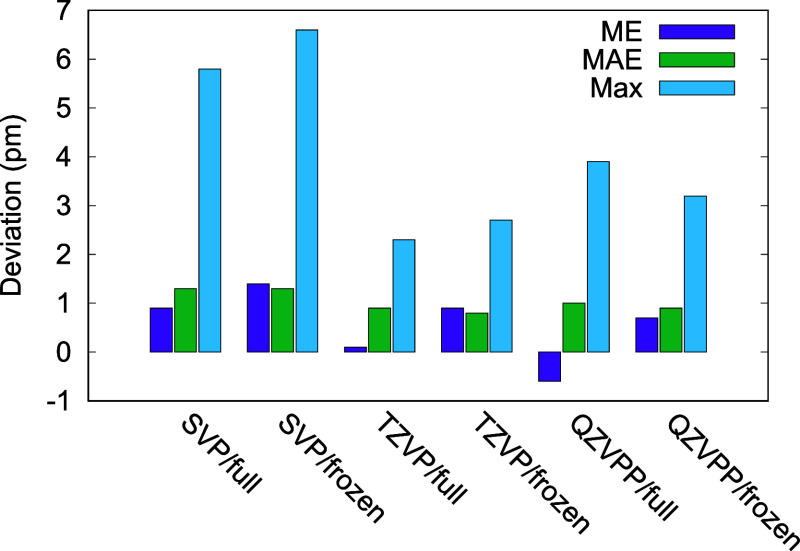
Mean deviation (ME), mean absolute deviation (MAE), and absolute maximum deviation (Max) for selected bond lengths (in pm) of a set of first-row transition metal complexes. Full-core (full) and frozen-core (frozen) RPA results for various basis sets are compared to the experimental bond lengths.^60^ SVP, TZVP, and QZVPP stand for def2-SVP, def2-TZVP, and def2-QZVPP basis sets, respectively.

The performance of analytical RPA gradients has previously been compared with a set of first-row transition metal complexes for which accurate experimental geometric data are available.^60^ Here, we reoptimize the complexes using the frozen-core RIRPA implementation and assess the difference with the full-core optimized structures. The results are shown in Figure 1 – see the Supporting Information for results on individual systems. The mean absolute deviation for all three basis sets used changes by less than 0.1 pm when freezing core orbitals. Changes of up to 1 pm are observed for the mean deviation with the largest change for the def2-QZVPP basis set. In line with the results for the main-group systems of Table 2, freezing core orbitals results in longer bond lengths on average. For all basis sets, the change in average bond length from full-core to frozen-core is smaller in magnitude than the average deviation from the experimental values. This supports the conclusion that for RPA geometry optimizations the frozen-core option can be used without significant loss of accuracy and furthermore affirms the finding from previous work that for RPA structures a triple-ζ basis set suffices.^12^ It should be noted that the Karlsruhe basis sets are not optimized for the core region and may therefore not completely capture all core–core and core–valence correlation. A full study of the impact of core–core and core–valence correlation on molecular geometries is left for future work.

#### Open-Shell Complexes

One of the main advantages of our implementation is the immediate applicability to both spin-restricted and unrestricted systems. Transition metal complexes are perfect examples of the need for such flexibility since their spin-states are often directly influenced by their coordination environment. The optimized ground state geometries for a small set of octahedral, first-row transition metal complexes were computed using def2-TZVPP and def2-QZVPP basis sets. PBE and PBE0 are reported for comparison. As noted previously,^28^ MP2 is not suitable for such systems and often yields metal–ligand bond lengths that are too short in comparison to a high-level theory reference or experimental data. Rather than focus on the absolute accuracy of RPA for these complexes, our goal is to assess the impact of the frozen-core option assuming that RPA’s performance is still comparable or superior to semilocal functionals.

As can be seen in Table 3, the metal–ligand distances predicted by RPA are similar to those predicted by PBE and PBE0. The frozen-core option generally increases the metal–ligand bond lengths by 0.5–2 pm, which is similar to the impact that the frozen-core makes on main group compounds calculated with MP2.^35^ Comparing the vibrational frequencies obtained for the full- and frozen-core options with def2-TZVPP basis sets, the differences range from a few wavenumbers to ∼10 cm^–1^ at most for these complexes, but due to the presence of both over and underestimates the RSMD is just a few wavenumbers for the frozen-core option.

**Table 3:** Optimized Metal-Ligand Distances (pm) Using def2-QZVPP Basis Sets for a Set of Octahedral Transition Metal Complexes with Varying Numbers of Unpaired Electrons^a^

					RIRPA		
	mult.	bond type	PBE	PBE0	full-Core	frozen-core	ω̃ RMSD
[V(urea)_6_]^3+^	3	(V–O)_av_	203.90	202.10	199.87	201.08	2.7
[Cr(NH_3_)_4_Cl_2_]^1+^	4	(Cr–N)_av_	212.92	211.36	208.38	209.75	3.8
		(Cr–Cl)_av_	227.91	227.34	225.87	226.91	
[Fe(H_2_O)_6_]^3+^	6	(Fe–O)_av_	207.04	203.92	202.54	203.56	4.6
[Co(NH_3_)_6_]^3+^	1	(Co–N)_av_	202.96	200.77	201.41	201.99	4.1
[Ni(en)_3_]^2+^	3	(Ni–N)_av_	218.09	217.20	214.13	215.56	3.8

a The root-mean-squared deviation (RMSD) between the RIRPA vibrational frequencies calculated with full- and frozen-core using def2-TZVPP basis sets is also reported. Average distances are reported since symmetry is not enforced during the optimization. mult = spin multiplicity, en = ethylenediamine.

#### Triferrous Extended Metal Atom Chain

To push the analysis to larger system size, a triferrous extended metal atom chain (EMAC) complex, Fe_3_L_3_, was recently characterized using all electron RPA calculations.^16^ The compound, Figure 3, contains 114 atoms with a linear triferrous core that adopts a high-spin (*S* = 6) ground state which retains *C*_*2*_ symmetry in the crystal structure. To compare with the results reported in ref (16)., def2-TZVP basis sets were used for all atoms, and the TPSSh functional was used to generate the reference determinant in C_1_ symmetry. Of particular interest in this compound are the Fe–Fe bond lengths, the metal–ligand distances characterized by an average Fe–N distance, and the average dihedral angle along the pyridine L^2–^ ligand, Table 4. The full-core calculations were previously carried out with 80 frequency grid points to ensure a small sensitivity measure, while the frozen-core calculation required only 40 frequency points to achieve a similar sensitivity measure.

**Table 4:** Geometric Parameters for Fe_3_L_3_ Calculated with RIRPA@TPSSh with def2-TZVP Basis Sets for All Atoms^a^

	avg Fe–Fe distance (Å)	avg Fe–N distance (Å)	avg L^2–^ dihedral angle (°)
frozen-core	2.480	1.990	46.3
full-core	2.473	1.984	47.0
crystal	2.442	1.985	45.5

a The frozen-core option yields excellent agreement with the full-core results, as well as with the experimental crystal structure.

Since symmetry constraints are not enforced for the RIRPA geometry optimization, a slight asymmetry develops in the Fe–Fe bond lengths, which amounts to less than 0.25 pm and is why the average is used instead. As seen previously for main group compounds, the frozen-core Fe–Fe and Fe–N bond lengths in Fe_3_L_3_ tend to elongate by less than 1 pm, while the dihedral angles are within 1 degree of the full-core result. To get a fuller sense of the differences in the other structural features, the RMSD of the optimized structures with full- and frozen-core was calculated to be 0.061 Å, which is acceptably small to consider the two structures close to one another. The error of the frozen-core option is smaller than the method error of RIRPA for this molecule with the chosen basis sets when compared to the crystal structure, and reinforces the ability of RPA to serve as a higher-level check on semilocal results for more strongly correlated systems, even when utilizing the frozen-core option. Timings for this molecule are discussed below.

### Timings

With the reduced sizes of some of the matrices used in the frozen-core calculation, there should be a speedup compared to the full-core calculation for a fixed number of frequency grid points. On the other hand, one of the major advantages of the frozen-core option is the ability to choose a smaller number of frequency grid points while maintaining a similar sensitivity measure to the full-core case with a Curtis-Clenshaw quadrature.^20^ Drontschenko et al. reported a speedup of 20–30% in their implementation^30^ for linear alkanes and a DNA fragment, depending on system and basis set size, with a fixed imaginary time/frequency integration grid. Here we report the impact of the frozen-core option with a fixed grid, as well as for a grid that targets a similar sensitivity measure as the full-core calculations. For the linear alkanes, molecules with 10–50 carbons were used to calculate the RIRPA gradient with def2-TZVP basis sets. Input structures for these calculations were obtained using CREST^61^ to find a minimum energy structure. In addition to these tests, the Fe_3_L_3_ molecule and a previously studied bipalladium complex^14^ were also used to asses the speedup of the frozen-core option.

**Figure 2:**
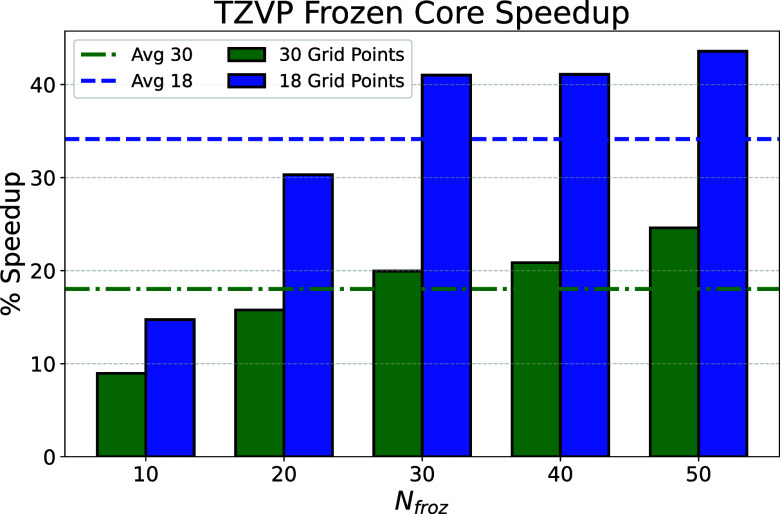
Percent speedup and average speedup for the frozen-core algorithm for a set of linear alkanes compared to the full-core implementation using 30 grid points with def2-TZVP basis sets. Using an equal number of grid points in the frozen-core case leads to ∼20% speedup on average, while targeting a similar sensitivity measure (18 grid points) yields larger speedups with an average closer to 35%.

The speedups for the alkanes with TZVP basis sets, and the average speedups, are plotted for the same grid size as the full-core calculation (30 points) and with a smaller grid (18 points), Figure 2. These calculations were carried out using a single thread on an Intel Xeon CPU (E5–2630 v3) using 32 GB of 2400 MHz RAM and a 7200 rpm SATA hard drive. For 30 grid points, the average sensitivity measure is approximately 1 × 10^–5^ for the full-core calculations, while it is closer to 9 × 10^–8^ for the frozen-core calculations. If the number of grid points is lowered to achieve a similar sensitivity measure as the full-core calculation, such as 18 grid points, then the frozen-core speedup is even larger due to the reduced size of the frequency grid, without sacrificing the accuracy of the analytical gradient. The reduction in grid size has a similar impact on the speedup as the frozen-core algorithm itself, therefore the combination of these two yields an average speedup of ∼35%.

**Figure 3:**
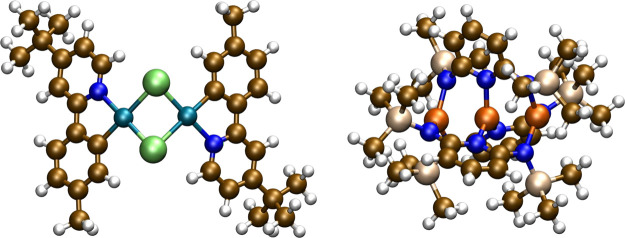
Chlorine-bridged dipalladium complex taken from Hansen et al.^62^ (left) and a triferrous complex taken from Bates et al.^16^ (right).

For the Fe_3_L_3_ complex, Figure 3, the 114 atoms generate 486 total electrons yielding 249 α and 237 β occupied orbitals. Using a 1s frozen core for C and N, a 1s2s2p frozen core for Si, and a 1s2s2p3s frozen core for Fe yields 90 core orbitals for each spin. This reduces the total number of occupied orbitals from 486 to 306, which is approximately a 35% reduction in the number of correlated electrons. With def2-TZVP basis sets, the total number of virtual orbitals is ∼1800. Using 12 threads on an Intel Gold 6252 2.1 GHz CPU, with a 12Gbps SAS solid state drive and 48 GB of 2933 MHz RAM, the total time for the full-core calculation using 80 frequency grid points was 12 h 36 min and 47 s. Using the same frequency grid, the frozen-core calculation required 7 h 37 min and 49 s, which is a 40% reduction in computational cost due to the frozen-core option. The sensitivity measure for the full-core calculation is close to 4 × 10^–6^ while the frozen-core calculation is close to 1 × 10^–8^ with 80 grid points. If the number of grid points is reduced to 40, then the frozen-core sensitivity measure increases to 1 × 10^–5^ and the total wall-time reduces to 6 h 38 min and 17 s, which is approximately a 47% speedup. As shown above, the frozen-core option minimally impacts the optimized structure, and as shown here can significantly reduce the computational costs for analytical RIRPA gradient calculations in large systems.

To further assess the increase in computational efficiency we optimized the geometry of a bipalladium complex consisting of 74 atoms, Figure 3, which was previously^14^ used to demonstrate the accuracy of RPA for reaction energies involving this complex. Efficient optimization of structures for complexes of this size further widens the scope of applicability of the RPA method. The complex was optimized using def2-TZVPP basis sets and self-consistent PBE orbitals with 70 grid points for the Curtis-Clenshaw quadrature, both with and without frozen core electrons. The results are summarized in Table 5. Using the default frozen core settings in turbomole yields 46 frozen occupied orbitals and 110 active occupied orbitals, approximately a 30% reduction in correlated electrons. The speedup obtained compared to the full-core calculation is also ∼30%. The calculation time can be further reduced by choosing a smaller grid size, which is evidently permitted given the small sensitivity parameter of 1 × 10^–9^ for the frozen-core calculation. This results in a speedup of over 55%. Furthermore, the number of optimization cycles required to reach convergence is smaller when using the frozen-core option and is reduced from 27 for the full-core calculation to 6 for the frozen-core calculation on the small grid. For completeness and to further corroborate the findings for bond lengths it is noted that the Pd–Pd distance changes by less than 1 pm when using a frozen core.

**Table 5:** Grid Size (in Points), Wall Times for One Optimization Cycle, Sensitivity Parameters, Number of Optimization Cycles, and Pd–Pd Bond Lengths (in pm) for a Bi-palladium Complex Optimized with the RIRPA Method with and without a Frozen-Core^a^

core	grid size	wall-time	sens. parameter	opt. cycles	Pd–Pd distance (pm)
full	70	2 h 42 min	10^–5^	27	311.92
frozen	70	1 h 55 min	10^–9^	18	312.44
frozen	30	1 h 11 min	10^–6^	6	312.43

a Results were obtained using 32 threads on an Intel(R) Xeon(R) CPU E5-2650 v4 @ 2.20 GHz processor with 132Gb of RAM and 340Gb of local scratch space on a 480GB Intel DC S3510 2.5” SATA 6Gbps MLC SSD.

## Conclusions

The frozen-core option has been implemented in combination with an extended Lagrangian for the random phase approximation leading to a computational speedup compared to the full-core implementation. Intermediate quantities built during the calculation have reduced dimension as a consequence of the restricted sums over active occupied orbitals leading to reduced memory and storage requirements as well. Furthermore, smaller integration grids can be utilized with the frozen-core option to achieve similar sensitivity measures to the full-core case with Curtis-Clenshaw quadratures, without loss of accuracy in the molecular properties. By adding the handling of an additional frozen-active occupied-occupied Lagrange multiplier, the full- and frozen-core algorithms are very similar when using the extended Lagrangian approach. Compared to the full-core implementation, the frozen-core option delivers a speedup of at least 40% while maintaining a high degree of accuracy for molecular geometries, dipole moments, and vibrational frequencies. Results for small, main-group molecules, as well as some prototypical 3*d* transition metal compounds, indicate that the frozen-core option yields bond lengths within a few picometers and vibrational frequencies that are typically within 10 cm^–1^ of the full-core values. These errors are much smaller than the RPA method errors and therefore encourage the use of the frozen-core option with RPA for efficient calculation of molecular properties based on nuclear gradients.

## Appendix

### Derivation of Lagrange Multipliers **W** and **D**^Δ^

To derive the equations for the various Lagrange multipliers needed in the frozen-core case, one can follow the same logic steps as originally presented in Appendix A in ref (28), but with a few modifications to account for the frozen-core. The expressions for the active–active occupied and virtual–virtual blocks of **T** follow directly from the application of eqs 53–59 using only the active occupied orbitals as described above. For the full occupied-virtual and active-frozen occupied blocks of **D**^Δ^ the orbital derivatives of the RIRPA Lagrangian must be modified to properly account for the frozen occupied orbitals. Following the notation of ref (28), ∑_μ_(∂/∂*C*_μ*p*σ_)_stat_*C*_μ*q*σ_≡*′*, the general orbital derivative can be expressed as

38

The first term corresponds to the derivative of the HF energy which explicitly contains contributions from both the frozen and active occupied orbitals, therefore

39

where δ is the Kronecker delta. The second term is directly related to a derivative of the correlation energy, which is restricted to just the active occupied orbitals, leading to a modified definition of Eq. (63) where *k* only runs over active occupied orbitals. This produces eqs 25 and 26 above for **γ**.

The derivative of the Lagrange multiplier **ε** contains contributions from both the frozen and active occupied orbitals leading to

40

which now contains contributions from **T**, **Z** and **Z̃** in the frozen-core case. Note that the frozen–frozen block of **D**^Δ^ is explicitly zero since these orbitals are not included in the correlation treatment. The last term in eq 38 remains formally unchanged from eq 67 in ref (28).

Combining these relationships together yields the needed equations to determine the different blocks of the Lagrange multiplier **W** from eq 38. Since the frozen and active occupied orbitals must be distinguished, there are now seven distinct blocks of this matrix that must be derived: frozen–frozen, active-frozen, frozen-active, and active–active occupied blocks, as well as all occupied-virtual, virtual-all occupied, and virtual–virtual blocks.

41

42

43

44

45

46

47

Since **W** should be symmetric at the stationary point, the equations for the off-diagonal blocks can be equated in order to derive the equations that determine the active-frozen and occupied-virtual **Z**-vectors. For the active-frozen occupied block one obtains

48

where the **ε**^HF^ and (**H**^+^**D**^Δ^) contributions cancel because they are symmetric. Assuming canonical orbitals, solving this equation for **Z̃** yields

49

Once **Z̃** has been obtained, only *W*_*if*σ_*or W*_*fi*σ_ is needed to finish computing the analytic gradient.

The same procedure can be applied to the full occupied-virtual blocks leading to modified forms of these equations compared to the full-core case. The major differences result from the appearance of **Z̃** and the restricted nature of **γ**,

50

In order to obtain eq 33 in ref (28), the contributions from **Z** must be separated from **T** and **Z̃** in the **D**^Δ^ term, leading to

51

where

52

and γ_*la*σ_ is defined in eq 29 above. Once **Z̃** and **Z** have been computed, the final gradients require only 5 blocks of **W** since the off-diagonal blocks will now be equivalent by construction to enforce the symmetry: *W*_*fg*σ_, *W*_*if*σ_, *W*_*ij*σ_, *W*_*al*σ_, and *W*_*ab*σ_.

Lastly, following the notation in Weigend and Häser,^35^ the various occupied contributions to **W** in the frozen-core case can be patched together to produce a matrix that has the same occupied-occupied dimension as the full-core case

53

leading to eq 30 above. In our current implementation, the additions between subspaces are done *after* transforming to the AO basis (eq 37) so that efficient linear algebra subroutines can be used to add matrices of the same dimension, see Figures S1 and S2 in the Supporting Information.

## Supporting Information Available

Outline of the frozen-core RIRPA gradient algorithm; numerical results for vibrational frequencies and dipole moments of main group compounds; and numerical results for first-row transition metal compounds (PDF)
Numerical results for vibrational frequencies of octahedral transition metal complexes (XLSX)
