# Supplementary material for: Frozen-Core Analytical Gradients within the Adiabatic Connection Random-Phase Approximation from an Extended Lagrangian
Source: J Chem Theory Comput. 2025 Mar 6;21(6):2977–87. doi: 10.1021/acs.jctc.4c01731 (PMC11948324; doi:10.1021/acs.jctc.4c01731)
Supplement: Supplementary file 1 — ct4c01731_si_001.pdf [file ct4c01731_si_001.pdf]

Supplemental Information for "Frozen-Core Analytical Gradients within the Adiabatic Connection Random Phase Approximation from an Extended Lagrangian"

Jefferson E. Bates[*,†] and Henk Eshuis[*,‡]

†*Department of Chemistry & Fermentation Sciences, Appalachian State University, Boone, North Carolina 28608-2021, United States*

‡*Department of Chemistry & Biochemistry, Montclair State University, Montclair, NJ 07043, United States*

E-mail: batesje@appstate.edu; eshuish@montclair.edu

Supporting Information Available

The Supporting Information is organized as follows. Results for main group compound dipole moments and vibrational frequencies are presented in Tables S1 and S2. Two figures are presented that summarize the algorithm for calculating analytic, frozen-core RIRPA gradients, Figures S1 and S2. Explicit results for bond lengths of first row transition metal compounds are presented in Table S3. Vibrational frequencies (in cm^{-1}) for the five octahedral, first-row transition metal complexes can be found in the tm_oct_vib_rirpa.xslx file included with the supporting information. These vibrational frequencies were calculated using central differences numerical differentiation, with a step size of 0.005 a.u.

(1) Canonical $\phi_{p\sigma}$, $\varepsilon_{p\sigma}$ from KS-SCF calculation
(2) $\Delta_{ia\sigma} \leftarrow \varepsilon_{a\sigma} - \varepsilon_{i\sigma}$ for $i \in$ active
(3) Compute Λ_{PQ}, Λ^{-1}_{PQ}
(4) Compute $B_{if\sigma,P}$, $B_{ij\sigma,P}$, $B_{ia\sigma,P}$ and store on disk in blocks I (Ref. 1)
(5) Determine frequency grid points ω_α and weights w_α (Ref. 2)
(6) Unrelaxed density matrix $\mathbf{T}$, accumulate $\mathbf{GB\tilde{Q}}$ on disk.
Loop ω_α
Loop σ
Loop I $\in$ active (subset of i)
$G_{ia\sigma}(\omega_\alpha) \leftarrow \Delta_{ia\sigma}/(\Delta^2_{ia\sigma} + \omega^2_\alpha)$
$\tilde{Q}_{PQ}(\omega_\alpha) \leftarrow 2B_{ia\sigma P} G_{ia\sigma}(\omega_\alpha) B_{ia\sigma Q}$
Compute Cholesky factor $\mathbf{L}(\omega_\alpha)$ of $(\mathbf{1} + \mathbf{Q}(\omega_\alpha))^{-1}$
$\tilde{\mathbf{Q}}_{PQ}(\omega_\alpha) \leftarrow L^{-1}_{PR}(\omega_\alpha) L^{-1}_{RQ}(\omega_\alpha) - \delta_{PQ}$
$\mathsf{QQt}_{PQ} \leftarrow -(\omega_\alpha/4\pi) Q_{PR}(\omega_\alpha) \tilde{Q}_{RQ}(\omega_\alpha)$ (for $\mathbf{\Gamma}^{(2)}$)
Loop σ
Loop I $\in$ active (subset of i)
$\mathsf{GBQt}_{ia\sigma P}(\omega_\alpha) \leftarrow G_{ia\sigma}(\omega_\alpha) B_{ia\sigma Q} \tilde{Q}_{QP}(\omega_\alpha)$
$\mathsf{Int2GBQt}_{ia\sigma P} \leftarrow 2(\omega_\alpha/2\pi) \mathsf{GBQt}_{ia\sigma P}(\omega_\alpha)$
$T_{ab\sigma} \leftarrow -[\mathbf{GBQt}(\omega_\alpha)\mathbf{B}^T\mathbf{G}(\omega_\alpha)]_{ia\sigma ib\sigma}$
$T_{ab\sigma} \leftarrow \omega^2_\alpha [\mathbf{\Delta}^{-1}\mathbf{GBQtB}^T\mathbf{G}\mathbf{\Delta}^{-1}]_{ia\sigma ib\sigma}$
Loop J $\leq$ I for J $\in$ active (subset of j)
$T_{ij\sigma} \leftarrow [\mathbf{GBQt}(\omega_\alpha)\mathbf{B}^T\mathbf{G}(\omega_\alpha)]_{ia\sigma ja\sigma}$
$T_{ij\sigma} \leftarrow -\omega^2_\alpha [\mathbf{\Delta}^{-1}\mathbf{GBQtB}^T\mathbf{G}\mathbf{\Delta}^{-1}]_{ia\sigma ja\sigma}$
(7) $\boldsymbol{\gamma}$ and $\mathbf{\Gamma}$ terms
Loop σ
Loop I $\in$ active (subset of i)
$\mathsf{GL}_{ib\sigma P} \leftarrow \mathsf{Int2GBQt}_{ib\sigma Q} \Lambda^{-1}_{PQ}$
$\mathsf{GC}_{i\nu\sigma P} \leftarrow \mathsf{GL}_{ib\sigma P} C_{\nu b\sigma}$
$\gamma_{ia\sigma} \leftarrow \mathsf{GC}_{i\nu\sigma P}(\mu\nu|P) C_{\mu a\sigma}$
$dE/d\xi \leftarrow \mathsf{GC}_{i\nu\sigma P} C_{\mu i\sigma} * d\Pi^{(3)}_{\mu\nu P}/d\xi$ ($\mathbf{\Gamma}^{(3)}$ term)
Loop J $\in$ active (subset of j)
$\gamma_{ij\sigma} \leftarrow \mathsf{GC}_{i\nu\sigma P}(\mu\nu|P) C_{\mu j\sigma}$
Loop F $\in$ frozen (subset of f)
$\gamma_{if\sigma} \leftarrow \mathsf{GC}_{i\nu\sigma P}(\mu\nu|P) C_{\mu f\sigma}$
$\tilde{Z}_{if\sigma} \leftarrow \gamma_{if\sigma}/2(\varepsilon_{i\sigma} - \varepsilon_{f\sigma})$
$\gamma_{ab\sigma} \leftarrow \mathsf{Int2GBQt}_{ia\sigma P} B_{ib\sigma P}$
$\gamma_{aj\sigma} \leftarrow \mathsf{Int2GBQt}_{ia\sigma P} B_{ij\sigma P}$
$\gamma_{af\sigma} \leftarrow \mathsf{Int2GBQt}_{ia\sigma P} B_{if\sigma P}$
$\gamma_{al\sigma} \leftarrow \gamma_{ai\sigma} \oplus \gamma_{af\sigma}$ for $i \in$ active, $f \in$ frozen, $l \in$ occupied
$\gamma_{la\sigma} \leftarrow \mathbf{0}_{fa\sigma} \oplus \gamma_{ia\sigma}$ for $f \in$ frozen, $i \in$ active, $l \in$ occupied
⋮

Figure S1: First part of the RIRPA analytic gradients algorithm.

$\vdots$

(8) $\mathbf{\Gamma}^{(2)}$ term

$\Gamma^{(2)}_{PQ} \leftarrow \Lambda^{-1}_{PR} \mathsf{QQt}_{RS} \Lambda^{-1}_{QS}$

$dE/d\xi \leftarrow \Gamma^{(2)}_{PQ} * d\Pi^{(2)}_{PQ}/d\xi$

(9) Right-hand side $\mathbf{R}$

$\tilde{T}^{\mathrm{AO}}_{\mu\nu\sigma} \leftarrow C_{\mu i\sigma} T_{ij\sigma} C_{\nu j\sigma} + C_{\mu a\sigma} T_{ab\sigma} C_{\nu b\sigma}$ for $i, j \in$ active

$\tilde{T}^{\mathrm{AO}}_{\mu\nu\sigma} \leftarrow C_{\mu i\sigma} \tilde{Z}_{if\sigma} C_{\nu f\sigma} + C_{\mu f\sigma} \tilde{Z}_{if\sigma} C_{\nu i\sigma}$ for $i \in$ active, $f \in$ frozen

$R^{\mathrm{AO}}_{\mu_\nu\sigma} \leftarrow H^{+,\text{AO-RI}}_{\mu\nu\sigma\kappa\lambda\sigma'} \tilde{T}^{\mathrm{AO}}_{\kappa\lambda\sigma'} + 2F^{\text{HF-RI}}_{\mu\nu\sigma}$

$R_{la\sigma} \leftarrow C_{\mu l\sigma} R^{\mathrm{AO}}_{\mu\nu\sigma} C_{\nu a\sigma} + \gamma_{la\sigma} - \gamma_{al\sigma}$ for $l \in$ occupied

(10) Solve $\mathbf{Z}$ vector equation $\rightarrow \mathbf{Z}$

(11) Lagrange multiplier $\mathbf{W}$

$D^{\Delta,\mathrm{AO}}_{\mu\nu\sigma} \leftarrow C_{\mu l\sigma} Z_{la\sigma} C_{\nu a\sigma} + C_{\mu a\sigma} Z_{la\sigma} C_{\nu l\sigma}$ for $l \in$ occupied

$D^{\Delta,\mathrm{AO}}_{\mu\nu\sigma} \leftarrow C_{\mu i\sigma} T_{ij\sigma} C_{\nu j\sigma} + C_{\mu a\sigma} T_{ab\sigma} C_{\nu b\sigma}$ for $i, j \in$ active

$D^{\Delta,\mathrm{AO}}_{\mu\nu\sigma} \leftarrow C_{\mu i\sigma} \tilde{Z}_{if\sigma} C_{\nu f\sigma} + C_{\mu f\sigma} \tilde{Z}_{if\sigma} C_{\nu i\sigma}$ for $i \in$ active, $f \in$ frozen

$\tilde{W}^{\mathrm{AO}}_{\mu\nu\sigma} \leftarrow H^{+,\text{AO-RI}}_{\mu\nu\sigma\kappa\lambda\sigma'} D^{\Delta,\mathrm{AO}}_{\kappa\lambda\sigma'} + 2F^{\text{HF-RI}}_{\mu\nu\sigma}$

$W_{lm\sigma} \leftarrow C_{\mu l\sigma} \tilde{W}^{\mathrm{AO}}_{\mu\nu\sigma} C_{\nu m\sigma}$ for $l, m \in$ occupied

$W_{if\sigma} \leftarrow 1/2\gamma_{if\sigma} + \tilde{Z}_{if\sigma} \varepsilon_{f\sigma}$ for $i \in$ active, $f \in$ frozen

$W_{ij\sigma} \leftarrow 1/2\gamma_\sigma + T_{ij\sigma} \varepsilon_{j\sigma}$ for i, j in active

$W_{lm\sigma} \leftarrow W_{lm\sigma} \oplus W_{ij\sigma}$ for $i, j \in$ active, $l, m \in$ occupied

$W_{ab\sigma} \leftarrow 1/2\gamma_{ab\sigma} + T_{ab\sigma} \varepsilon_{b\sigma}$

$W_{al\sigma} \leftarrow 1/2\gamma_{al\sigma} + Z_{al\sigma} \varepsilon_{l\sigma}$ for $l \in$ occupied

$W^{\mathrm{AO}}_{\mu\nu} \leftarrow C_{\mu a\sigma} W_{al\sigma} C_{\nu l\sigma} + C_{\mu l\sigma} W_{al\sigma} C_{\nu a\sigma}$ for $l \in$ occupied

$W^{\mathrm{AO}}_{\mu\nu} \leftarrow C_{\mu i\sigma} W_{if\sigma} C_{\nu f\sigma} + C_{\mu f\sigma} W_{if\sigma} C_{\nu i\sigma}$ for $i \in$ active, $f \in$ frozen

$W^{\mathrm{AO}}_{\mu\nu} \leftarrow C_{\mu l\sigma} W_{lm\sigma} C_{\nu m\sigma} + C_{\mu a\sigma} W_{ab\sigma} C_{\nu b\sigma}$

(12) Add remaining contributions to gradients

$\tilde{D}_{\mu\nu\sigma} \leftarrow D_{\mu\nu\sigma} + D^{\Delta,\mathrm{AO}}_{\mu\nu\sigma}$

$dE/d\xi \leftarrow \tilde{D}_{\mu\nu\sigma} * dh_{\mu\nu\sigma}/d\xi$

$dE/d\xi \leftarrow (D_{\mu\nu\sigma} \tilde{D}_{\kappa\lambda\sigma'} - 1/2 D_{\mu\kappa\sigma} D_{\nu\lambda\sigma'} \delta_{\sigma\sigma'}) * d\Pi^{(4)}_{\mu\nu\sigma\kappa\lambda\sigma'}/d\xi$ ($\mathbf{\Gamma}^{(4)}$ term)

$dE/d\xi \leftarrow D^{\Delta,\mathrm{AO}}_{\mu\nu\sigma} * \partial V^{\mathrm{XC}}[\mathbf{D}]/d\xi$

$dE/d\xi \leftarrow W^{\mathrm{AO}}_{\mu\nu} * dS_{\mu\nu}/d\xi$

Figure S2: Second part of the RIRPA analytic gradients algorithm

Table S1: Dipole moments (debye) obtained using def2-QZVPPD basis sets at the optimized geometry for each method with RIRPA. The frozen-core option changes the dipole moment by less than 0.01 D. Experimental values taken from Ref. 3.

	Exp.	Frozen-core	Full-core
HF	1.826	1.752	1.745
CO	0.11	0.099	0.101
H_2O	1.85	1.809	1.812
pyrrole	1.767	1.838	1.844
furan	0.685	0.645	0.637

Table S2: Vibrational frequencies obtained using def2-QZVPPD basis sets at the optimized geometry for each method with RIRPA. The frozen-core option changes the dipole moment by less 1%. Reference values taken from Ref. 4 and 5.

	vibration	Ref.	Frozen-core	Full-core
HF	σ stretch	4138	4070	4093
CO	σ stretch	2170	2116	2133
H_2O	a_1 bend	1646	1649	1649
	a_1 stretch	3832	3768	3802
	b_1 stretch	3945	3873	3913

Table S3: Individual bond lengths for first row transition metal systems averaged in Figure 1. Reference values are taken from Bühl and Kabrede.[6] All other results are obtained using RIRPA method starting from self-consisten PBE orbitals either using all electrons (full) or a frozen-core (fc). Karlsruhe basis sets were used, where qzvpp is def2-QZVPP, tzvp is def2-TZVP, and svp is def2-SVP.

Compound	Distance	Ref Values	qzvpp/full	qzvpp/fc	tzvp/full	tzvp/fc	svp/full	svp/fc
$Sc(acac)_3$	Sc-O	207.6	207.7	208.7	208.91	209.43	209.2	209.27
$TiCl_4$	Ti-Cl	216.9	216.8	217.7	217.94	218.4	219.09	219.18
$TiMeCl_3$	Ti-C	204.7	203.8	204.9	205.6	206.27	207.44	207.63
	Ti-Cl	218.5	217.9	218.8	218.9	219.37	219.76	219.86
$TiMe_2Cl_2$	Ti-C	205.8	204.6	205.6	206.14	207.76	207.84	208.03
	Ti-Cl	219.8	219.3	220.1	220.16	220.64	220.77	220.85
VOF_3	V=O	157.0	158.2	158.5	158.15	158.61	157.2	157.55
	V-F	172.9	172.7	173.4	172.92	173.34	172.19	172.47
VF_5	V-Fax	173.4	175.2	175.7	175.25	175.61	175.6	175.71
	V-F eq	170.8	171.7	172.4	171.78	172.2	171.5	171.8
$VOCl_3$	V=O	157.3	158.2	158.3	158.13	158.56	157.1	157.47
	V-Cl	213.8	213.5	214.5	214.96	215.67	217.44	217.49
V(Cp)(CO)4	V-CCO	196.3	193.6	195.3	194.27	195.59	196.4	196.73
$V(NMe)_4$	V-N	187.9	187.2	188.0	187.49	188.31	188.33	188.72
CrO_2F_2	Cr=O	157.4	158.3	158.6	158.04	158.45	157.02	157.36
	Cr-F	171.9	171.3	172.3	172.2	172.6	171.86	172.08
CrO_2Cl_2	Cr=O	157.7	158.5	158.8	158.26	158.65	157.17	157.51
	Cr-Cl	212.2	210.8	212.6	213.1	213.76	216.97	216.97
$CrO_2(NO_3)_2$	Cr=O	158.4	159.0	159.3	158.68	159.11	157.22	157.55
	Cr-O	195.4	191.5	193.2	193.09	193.68	196.13	196.26
$Cr(C_6H_6)_2$	Cr-C	215.0	213.0	215.0	213.87	215.2	213.66	214.5
$Cr(C_6H_6)(CO)_3$	Cr-C Ar	220.8	219.2	220.2	219.35	220.5	218.91	219.73
	Cr-C co	186.3	185.8	186.7	185.8	186.88	187.63	187.78
$Cr(NO)_4$	Cr-N	175.0	175.4	176.2	175.72	176.39	175.16	175.66
MnO_3F	Mn=O	158.6	159.4	159.8	159.15	159.6	158.76	159.11
	Mn-F	172.4	171.0	172.1	172.36	172.71	173.15	173.34
$MnCp(CO)_3$	Mn-C Cp	214.7	213.6	215.3	214.59	215.55	214.35	215.3
	Mn-C Co	180.6	179.3	180.8	179.91	180.93	182.15	182.22
$Fe(CO)_5$	Fe-C mean	182.9	180.9	182.2	181.45	182.15	183.62	183.77
$Fe(CO)_3(tmm)$	Fe-C Co	181.0	179.3	180.7	179.75	180.67	181.72	182.03
	Fe-C cent	193.8	192.8	194.2	194.13	194.84	194.5	195.08
	Fe-C CH_2	212.3	210.3	211.7	211.54	212.51	210.46	211.18
$Fe(CO)_2(NO)_2$	Fe-C mean	187.2	184.5	186.2	185.42	186.38	188.12	188.24
	Fe-N	167.4	168.0	168.5	168.26	168.71	168.28	168.7
$FeCp_2$	Fe-C	206.4	203.9	205.9	204.5	205.64	204.95	205.62
$Fe(C_2H_4)(CO)_4$	Fe-C et	211.7	209.4	210.8	210.48	211.46	208.18	209.09
	Fe-C ax	181.5	180.6	181.8	181.24	181.87	183.4	183.6
	Fe-C eq	180.6	179.9	181.4	180.49	181.36	182.7	182.99
$Fe(C_5Me_5)(P_5)$	Fe-P	237.7	237.9	239.1	239.07	240.11	237.36	238.35
$CoH(CO)_4$	Co-C eq	179.8	179.5	182.1	180.12	181.64	181.66	182.44
$Co(CO)_3(NO)$	Co-N	165.8	166.7	168.1	167.02	167.96	166.91	168.43
	Co-C	183.0	182.3	185.4	183.12	184.78	185.4	186.01
$Ni(CO)_4$	Ni-C	182.5	182.6	185.6	183.56	185.15	184.62	185.55
$Ni(acac)_2$	Ni-O	187.6	185.4	187.3	186.61	187.55	189.64	190.46
$Ni(PF_3)_4$	Ni-P	209.9	209.7	211.3	210.67	211.75	210.01	211.08
$CuCH_3$	Cu-C	188.4	188.4	191.6	189.53	190.79	194.18	194.97
CuCN	Cu-C	183.2	183.2	186.0	183.86	185.3	186.84	189.01
$Cu(acac)_2$	Cu-O	191.4	192.2	193.6	193.51	193.96	195.59	196.17

References

(1) Weigend, F.; Häser, M. RI-MP2: first derivatives and global consistency. Theor. Chim. Acta **1997**, 97, 331.

(2) Eshuis, H.; Yarkony, J.; Furche, F. Fast computation of molecular random phase approximation corelation energies using resolution of the identity and imaginary frequency integration. J. Chem. Phys. **2010**, 132, 234114.

(3) Adamo, C.; Cossi, M.; Scalmani, G.; Barone, V. Accurate static polarizabilities by density functional theory: assessment of the PBE0 model. Chem. Phys. Lett. **1999**, 307, 265 – 271.

(4) Ruden, T. A.; Helgaker, T.; Jørgensen, P.; Olsen, J. Coupled-cluster connected quadruples and quintuples corrections to the harmonic vibrational frequencies and equilibrium bond distances of HF, N2, F2, and CO. J. Chem. Phys. **2004**, 121, 5874–5884.

(5) A.R. Hoy, I. M.; Strey, G. Anharmonic force constant calculations. Mol. Phys. **1972**, 24, 1265–1290.

(6) Bühl, M.; Kabrede, H. Geometries of Transition-Metal Complexes from Density-Functional Theory. J. Chem. Theory Comput. **2006**, 2, 1282–1290.
